# Interactions between tall oatgrass invasion and soil nitrogen cycling

**DOI:** 10.1007/s00442-022-05192-x

**Published:** 2022-06-07

**Authors:** Eve-Lyn S. Hinckley, Hannah R. Miller, Ann Lezberg, Brian Anacker

**Affiliations:** 1 Institute of Arctic and Alpine Research, Sustainability, Energy, and Environment Complex, 4001 Discovery Drive, Boulder, CO 80303 USA; 2 Department of Environmental Studies, University of Colorado, Sustainability, Energy, and Environment Complex, 4001 Discovery Drive, Boulder, 80303 USA; 3 City of Boulder Open Space and Mountain Parks, 2520 55th Street, Boulder, CO 80301 USA

**Keywords:** Biogeochemistry, Invasion ecology, Nitrification, Nitrogen mineralization, Plant-soil interactions, Restoration, Species diversity, Tallgrass prairie

## Abstract

Increases in nitrogen (N) inputs to the biosphere can exacerbate the introduction and spread of invasive non-native plant species. Often, with elevated soil N levels, invasive plants establish and further enrich soil N pools, changing overall ecosystem function. This study examined the relationship between soil N cycling and an increasingly prevalent, invasive plant species, tall oatgrass (*Arrhenatherum elatius* subsp. *elatius*), in foothills ecosystems between the Colorado Rocky Mountains and the Denver-Boulder Metropolitan area—similar to many Western US grasslands and woodlands. It focused on investigating differences in soil N transformations, inorganic N pools, and vegetation characteristics across invaded and uninvaded plots at three sites in two seasons (summer and autumn). There was a statistically significant effect of invasion on rates of net N mineralization, but it was dependent on site and season (*p* = 0.046). Site had a statistically significant effect on soil moisture and aboveground biomass C:N (*p* < 0.04). The interactions of invasion x site were statistically significant for ammonium pools (*p* < 0.03). These findings suggest that *A. elatius* invasion can be associated with accelerated N cycling, but that the nature of the relationship differs by location and season in the foothills. More broadly, this study contributes to determining how the N cycle is shifting in grassland ecosystems subject to increasing pressures from anthropogenic change.

**Supplementary Information:**

The online version contains supplementary material available at 10.1007/s00442-022-05192-x.

## Introduction

Worldwide, increases in atmospheric N deposition to the biosphere have exacerbated the introduction and spread of invasive non-native plant species and lowered plant diversity (Ehrenfeld 2003; Davidson et al. 2011; Field et al. 2014). Prior research demonstrates that high soil N environments resulting from elevated N inputs can give invasive plants a competitive advantage (Dukes and Mooney 1999; Clark and Tilman 2008). In addition, invasive plants can affect soil N cycling via several mechanisms, including N fixation (e.g., Asner et al. 2008; Kurokawa et al. 2010), production of N-rich tissues (e.g., Kurokawa et al. 2010; Lee et al. 2017), and biomass growth (e.g., Aguilera et al. 2010). Often, a positive feedback ensues: the invasive continues to enrich the soil inorganic N pool, perpetuating conditions that favor its success or the success of other non-natives (Corbin and D’Antonio 2003; Liao et al. 2008; Shaben and Myers 2010), or changing plant species composition and accelerating N cycling (Bobbink et al. 2010). This interaction between elevated N inputs and plant species invasion can create notable changes in ecosystem function that make restoration activities challenging for land managers.

For the last several decades, the foothills and prairie ecosystems on the eastern slope of the Colorado Rocky Mountains have experienced the interacting effects of elevated atmospheric N deposition and changes to plant species composition due to invasive species introductions. Crawford and colleagues (2020) report that atmospheric N deposition as nitrate—once nearly double that of the less populated western slope (Baron et al. 2000)—is declining, likely due to air pollution regulation. However, ammonium-N inputs are increasing, which may be sourced from agricultural areas in Eastern Colorado. At the same time, the invasive perennial grass, tall oatgrass (*Arrhenatherum elatius* subsp. *elatius*), has replaced large areas once dominated by native prairie species (EnvironPlan Partners 2018). Originally introduced in the mid-nineteenth century by ranchers, spread of *A. elatius* was likely controlled by grazing. However, with cessation of grazing around the 1960s, as well as fire suppression and possibly elevated atmospheric N deposition levels, it spread rapidly, and its expansion continues today, making it the predominant plant species in invaded areas (EnvironPlan Partners 2018). During the last decade, land managers have been evaluating several approaches to contain *A. elatius*, including prescribed fire, pesticide applications, and grazing.

Previous research on the relationship between *A. elatius* invasion and the soil N cycle have focused on several factors related to structural and soil chemical effects of the invasive. For example, Berendese et al. (1992) found that areas receiving N fertilizer inputs within hayfields of the Netherlands favored persistence of *A. elatius* compared with unfertilized areas that were dominated by native red fescue (*Festuca rubra*). In addition, they found that clipping *A. elatius* caused more allocation of N to its aboveground tissues, which led to increased ecosystem losses of N, compared with uninvaded areas. Other studies determined that *A. elatius* increases rates of decomposition above even that of other aggressive invasive plant species (Holub et al. 2012).

The goal of our study was to determine if *A. elatius* is associated with an altered soil N cycle in sites where it is a serious threat to ecosystem health and integrity. Specifically, we addressed the question: what is the nature of the relationship between *A. elatius* and the soil N cycle in foothills and grassland ecosystems? Given that invasive plants often speed up N cycling through high quality litter and rapid growth, we hypothesized that *A. elatius* would be associated with higher soil inorganic N (ammonium and nitrate). In addition to evaluating differences in net N cycling rates, we also measured other ecosystem factors that might affect N cycling rates (soil moisture) or change in response to invasion (aboveground biomass and aboveground biomass C:N). Our study is a first step to inform future efforts that address the interactions among *A. elatius*, soil N cycling, and management practices. More broadly, it can provide a foundation for conducting process-based study of the relationships among atmospheric N deposition, soil N cycling, and ecosystem functioning from the plains to the alpine.

## Methods

### Study area

We conducted this research at three grass-dominated study sites in the foothills of the Colorado Front Range, all managed by Boulder Open Space and Mountain Parks (OSMP) in Boulder, Colorado, US (Fig. S1, Table 1). We chose three sites to represent some of the different vegetation structural types in which *A. elatius* grows across the foothills area. We refer to the sites with labels that represent the growth form of the tallest layer of vegetation at each site. These sites include: “shrubland”, located in Coyote Canyon, a steep ravine with north- (invaded) and south-facing (largely uninvaded) slopes supporting a mix of scattered shrubs and grass-dominated herbaceous vegetation (Fig. S1a); “grassland”, located south of the National Center for Atmospheric Research (NCAR) mesa where mixed grass prairie has been invaded by broad swaths of dense *A. elatius*, the highest percent cover observed in this study (Fig. S1b); and “savanna”, located on Shanahan Ridge in an open woodland area with dense patches (~ 5–10 m) of *A. elatius* invasion interspersed with other grasses and trees (Fig. S1c).

**Table 1:** Location information and soil characteristics (mean ± 1 SE) of uninvaded plots

Site	Slope (%)	Soil type^1^	Bulk density (g cm^−3^)	Soil pH^2^	Soil C:N^2^	Soil N (%)^2^
Grassland	15	Colluvial land, gravelly sandy loam	0.89	6.69 ± 0.17	13.5 ± 1.1	0.40 ± 0.01
Shrubland	28	Colluvial land, gravelly sandy loam	1.02	6.25 ± 0.60	17.0 ± 1.4	0.24 ± 0.04
Savanna	1	Nederland very cobbly sandy loam	0.83	6.17 ± 0.21	15.4 ± 2.7	0.34 ± 0.04

^1^Soil type determined from NRCS survey area (version 17, 5 June 2020)

^2^*n* = 3 per site

Average monthly air temperature in the Denver-Boulder Metropolitan area ranges from approximately 0 °C in winter to 21 °C in summer and mean annual precipitation to the foothills of the Colorado Front Range is ~ 790–890 mm (Wetherbee et al. 2019).

### Study design

At each of three study sites, we established three 2 m × 2 m plots within *A. elatius*-invaded and uninvaded areas, respectively (*n* = 18 across sites and treatments). The patchiness of *A. elatius* invasion precluded random plot location. Thus, we chose plot locations based on the following criteria: invaded plots were placed within patches of 40–80% *A. elatius* cover, and uninvaded plots were located at least 2 m from the nearest *A. elatius* individual, with 0% *A. elatius* cover within the plot. At the grassland and savanna sites, the plots were placed in uninvaded and invaded areas along a common slope and aspect. At the shrubland site, plots were located along two transects parallel to the slope.

### Field sampling

Each site was sampled two times during the study period (Sept 2019–Oct 2020) for soil N processing rates, inorganic N pools, and gravimetric soil moisture. We targeted two key phenological seasons to make these measurements: Summer, Jun–Jul 2020, to capture peak biomass, and autumn, Sept–Oct 2020, to measure plant senescence and seasonal transition. For each sampling period, paired soil cores (3 cm diameter × 10 cm depth) were collected at each plot, roots were removed (to exclude plant N uptake), and the cores were placed in plastic bags, one for immediate analysis of soil inorganic N species, and the other returned to its borehole for incubation in the field, following the in-field buried bag technique described by Hart et al. (1994). Both cores were extracted for inorganic N concentrations and then the rates of net N cycling processes were calculated by differencing the results of the final, incubated core and the initial core (see “Data Analysis”). Following incubation (~ 30 days), soil cores were transported to the laboratory for analysis in the same manner as the ones subjected to immediate analysis (see description below). Once during the study period, we collected soil cores for soil C:N, soil pH, and bulk density (Sept 2019, and Sept and Oct 2020, respectively).

In addition to soil measurements, we sampled vegetation within each plot for aboveground biomass, C:N ratios, and *A. elatius* abundance (as percent aerial cover). We collected samples from 0.5 m × 0.5 m quadrats (one per plot) in Sept 2019. Standing stems plus leaves and litter (“thatch”) cover were clipped to the ground surface within the quadrat; it is important to note that these collections occurred when *A. elatius* had senesced at the end of summer/early autumn. In the laboratory, aboveground biomass samples were dried at 60 ºC for 48 h, then weighed to determine dried biomass per unit area. After cutting and mixing each sample, a homogeneous subsample of aboveground biomass tissue was pulverized with a mortar and pestle and analyzed for total C and N, as described below. We estimated plant species cover within a 1 m^2^ area of each plot using visual cover and then identified the presence of all remaining species within the remaining 4 m^2^ plot. Dominant plant species were any species that had > 20% areal cover.

### Laboratory analysis

Immediately upon return from the field, soil cores were hand-picked to remove roots and rocks. Subsamples of field-moist soil were extracted in 2 M potassium chloride (KCl), shaken for 2 h, and then filtered using Whatman 1 filters, modified from Binkley et al. (1986) and Hart et al. (1994). Extractants were analyzed for ammonium and nitrate concentrations with a Lachat QuikChem 8500 Flow Injection Autoanalyzer, detection limit of 0.005 mg NH_4_^+^–N L^–1^ and 0.004 mg NO_3_^−^–N L^–1^, respectively. In addition, a subsample of each soil sample was analyzed for gravimetric soil moisture (dried at 105 ºC for 48 h) and subsamples of oven-dried (60 ºC for 48 h) and ground soil and vegetation tissue were analyzed for total C and N by combustion on a Thermo Finnigan Flash EA 1112. All analyses were completed in the Arikaree Environmental Laboratory at University of Colorado, Boulder.

### Data analysis

We calculated the rate of net N mineralization by differencing the mass of inorganic N (ammonium) in final and initial soil extracts and dividing by the incubation period to get the mass of N produced per gram of dry soil per day for each of two seasons. We also calculated the rate of nitrification but it was highly correlated with mineralization (*r*^2^ = 0.94, *p* < 0.001), and, therefore, not analyzed further. Net N mineralization rate is reported on an areal basis using bulk density to scale. We fit three linear models, one per response variable—net N mineralization rates, soil ammonium pools, and soil moisture—to examine the relationship between *A. elatius* invasion and soil properties. Models share the same predictors (site, invasion, season, and their interactions) as fixed effects and have 36 observations. We fit two additional models for aboveground vegetation (aboveground biomass and C:N ratios). Finally, we created a structural equation model (SEM) to relate *A. elatius* cover to soil and vegetation variables simultaneously. Due to the limited nature of our sample size, we rely on the linear models for the interpretation of our results in the main text and provide the SEM results in the Supplementary Material.

We used *R* (version 1.2.5033) for all analyses, including the following packages: car, dplyr, forcats, ggplot2, MASS, semTools, semPlot, tidyr, and vegan. The complete dataset (10 variables, 36 observations) is included in the Supplementary Material (Table S1).

## Results

**Fig. 1:**
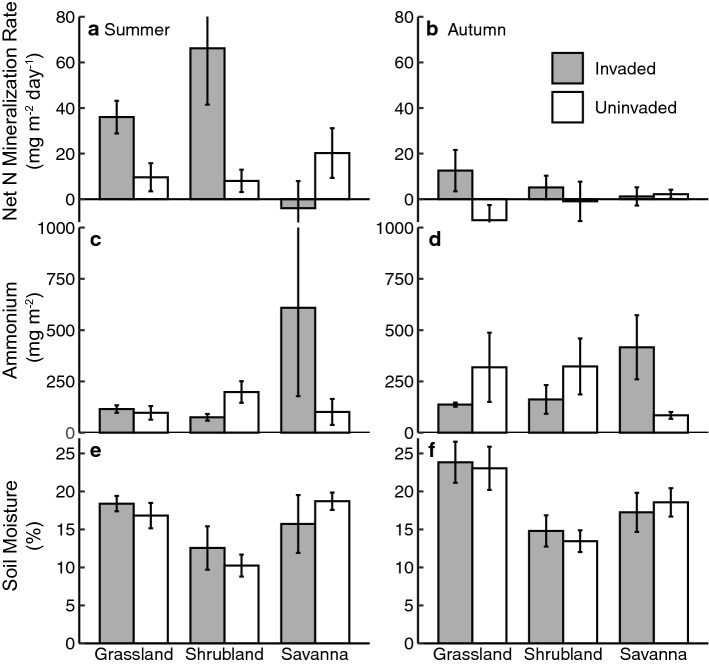
Comparisons of soil response variables across sites and seasons. Summer patterns are in panels **a**, **c**, and **e**, autumn patterns are in panels **b**, **d**, and **f**. *n* = 3 per treatment per site and bars are ± 1SE

The characteristics of the three sites (i.e., locations and soils) are summarized in Table 1. The linear models demonstrated that invasion by *A. elatius* was associated with higher rates of net N mineralization, but this result was dependent on site as well as season (site × invasion × season, *p* = 0.046; Fig. 1a; and 1b Table 2). Rates were highest in summer, in invaded plots, and at the grassland and shrubland sites; the savanna did not match our initial expectations and had lower net N mineralization in the invaded than the uninvaded plots. Soil ammonium pools were not well predicted (*r*^2^ = 0.06), but invasion was associated with lower ammonium pools in some contexts (site × invasion *p* = 0.028; Fig. 1c and 1d; Table 2). Soil nitrate pools were not significant relative to predictors (*P* = 0.094), and, thus, are not discussed further in detail.

**Table 2:** Statistical results for each model

Response variable	Invasion	Site	Season	Site × invasion	Site × season	Invasion × season	Site × invasion × season	*r*^2^
Net N mineralization	0.02*	0.15	0.002**	0.01*	0.16	0.35	0.046*	0.50
Ammonium	0.43	0.38	0.65	0.028*	0.50	0.43	0.91	0.06
Soil moisture	0.83	< 0.001**	0.03*	0.42	0.29	0.42	0.90	0.40
Aboveground biomass	0.06	0.43	–	0.60	–	–	–	0.28
Aboveground biomass C:N	0.48	0.03*	–	0.015*	–	–	–	0.49

*p < 0.05; **p < 0.01

**Fig. 2:**
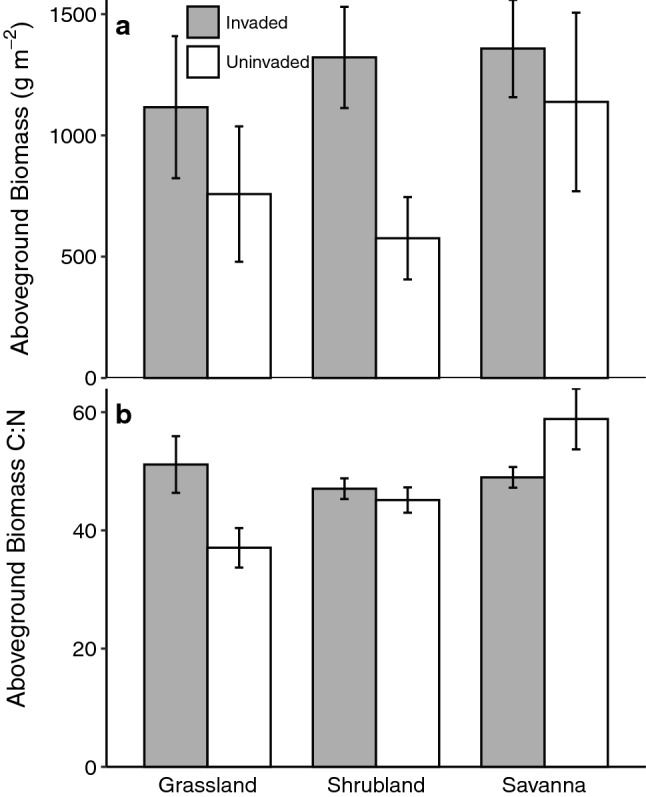
Comparisons of vegetation response variables across sites. **a** Aboveground biomass (leaves, stems, and thatch) and **b** C:N ratios in invaded and uninvaded plots. *n* = 3 per treatment per site and bars are ± 1 SE

Soil moisture was not significantly related to invasion, but site and seasonal differences were obvious (site *p* < 0.001; season *p* = 0.03; Fig. 1e and 1f; Table 2). Similarly, aboveground biomass was not significantly related to invasion (*p* = 0.06). This result was likely due to high variability among our plots, yet there was a pattern of higher average aboveground biomass in invaded plots at all three sites (Fig. 2a). Aboveground biomass C:N was higher in invaded plots at the grassland and shrubland sites, but not at the savanna site (site *p* = 0.03; site × invasion *p* = 0.015; Fig. 2b).

For the five linear models, we tested if each one met the assumptions of normality of residuals and homogeneity of variance. The assumptions were met for three of the five models. For the two offending models (soil ammonium pools and net N mineralization rates), model assumptions could be met via transformation of the data (i.e., square root or log transformation). In the case of ammonium pools, data transformation made no difference to the assessment of data significance. In the case of net N mineralization rates, data transformation changed the *p* value for the three-way interaction from 0.046 to 0.067. Upon inspection, the square root transformation that we used appeared to decrease the leverage of a single outlying data point indicating a very high net N transformation rate; this change allowed model assumptions to be met but nudged the *p* value above alpha 0.05. Nevertheless, for convenience, we share the results from using conventional linear models and untransformed data.

The SEM was difficult to fit (i.e., failed to converge) due to our small sample size and the large among-site differences. After removing the savanna site, we were able to get a model to converge; it showed that *A. elatius* cover was a direct, significant predictor of vegetation variables (*p* < 0.001), which was, in turn, a significant predictor of soil variables (*p* = 0.034). More information is presented as a supplement to our study, but not discussed further here.

## Discussion

This study addressed the question of whether *A. elatius*, an invasive grass in the Colorado Front Range, is associated with altered cycling of N, a nutrient that is often limiting to plant growth in terrestrial ecosystems. Elevated atmospheric N deposition from anthropogenic sources in the Denver-Boulder Metropolitan area contribute additional N inputs to ecosystems of the Colorado Front Range (Wetherbee et al. 2019; Crawford et al. 2020). We hypothesized that with greater abundance of *A. elatius*, soil N cycling would speed up—a common effect of invasive plant species, due to higher quality litter and rapid growth. While we know that *A. elatius* is not an N-fixing species, process-based studies of the plant-soil system that examine the degree to which the invasive is associated with altered soil N cycling (following Lee et al. 2017) have been largely unstudied. Our research was designed to explore this unknown.

We found that there was a significant trend toward higher rates of net N mineralization associated with invasion in two of the three sites that we studied (Fig. 1a and 1b); the grassland site, an open grassland area, and the shrubland site, a steep grassy ravine with regular shrub occurrence. We believe that the same pattern was not observed at the savanna site because plots were located across more variable soil and vegetation environments, including some under the canopy and overlying the litter of coniferous trees, which often have lower rates of net N cycling processes (Aber et al. 1991; Compton et al. 1998). In addition, there was a greater presence of other invasive species in both *A. elatius*-invaded and uninvaded plots (e.g., *Bromus japonicus*), which may influence soil N cycling and/or plant community dynamics at the savanna site. The net N rates that we observed are consistent with patterns in other ecosystems along the Colorado Front Range: at >  ~ 30 kg N ha^−1^ in two of the three invaded areas and >  ~ 9.6 kg N ha^−1^ in all uninvaded areas. Across the summer months alone, net N mineralization rates exceed atmospheric N deposition of ~ 4 kg N ha^−1^ yr^−1^ (e.g., Chen et al. 2020; Crawford et al. 2020). Contrary to our initial expectations, our findings highlight variable results across the three sites, regarding the association between *A. elatius* and an accelerated soil N cycle.

Although soil moisture levels—a first-order control on microbial activity—were significantly lower in summer than during the autumn sampling period (Fig. 1e and 1f; Table 2), microbes were active in the summer and released inorganic N in excess of their metabolic demands (i.e., > 0 mg N m^−2^ day^−1^), with more soil inorganic N released in invaded than uninvaded areas. Measurement of comparatively higher rates of net N mineralization in summer is consistent with patterns observed by McCulley et al. (2009) in short-, mixed, and tallgrass prairie ecosystems, as well as Owen et al. (2003) in a Taiwanese grassland ecosystem. However, it is contrary to Liu et al. (2010) who observed the lowest net N cycling rates during the growing season in temperate grasslands of Inner Mongolia.

In contrast, the lower rates of net N mineralization that we observed during autumn sampling indicate potential consequences for changes to the ecosystem N balance (Fig. 1b). During autumn, net N mineralization rates were at least three-fold lower than during the summer but remained positive (indicating release of inorganic N into the soil) in invaded areas (Fig. 1a and 1b; Table 2). However, in uninvaded areas, net N mineralization rates at the shrubland and grassland sites were lower and more likely to be negative—indicating net N immobilization—in autumn (Fig. 1b). A tendency toward low net N mineralization rates is consistent with previous studies in tallgrass prairie ecosystems (Risser and Parton 1982; McCulley et al. 2009). Our results suggest that the soil N cycle in uninvaded areas may be more closed when plants senesce in the autumn; that is, any inorganic N released by microbes is more likely to be immobilized in their tissues and remain within the soil. Yet microbes in *A. elatius*-invaded areas may still release inorganic N in excess of their metabolic demand. Inorganic N may be subject to other ecosystem fates (e.g., leaching) or remain in the soil, leaving it enriched for the start of the following growing season.

It is important to consider the potential fates of inorganic N released into the soil. We observed an effect of site × invasion on soil ammonium pools (*p* = 0.06), which provides some important insights about variation in soil N dynamics across Colorado Front Range grasslands. Overall, soil ammonium pools were within the range of those reported for comparable prairie ecosystems (McCulley et al. 2009). Interestingly, of the three sites, the savanna had the highest soil ammonium pools in both summer and autumn (Fig. 1c and 1d), with invaded plots associated with higher pools than uninvaded plots. Yet, the savanna also had lower net N mineralization rates than the other sites, particularly in invaded areas (Fig. 1a and b). In contrast, the grassland, the site with greatest percent cover of *A. elatius* in invaded areas and comparatively higher net N mineralization rates, had lower inorganic N pools overall (Fig. 1). There, invaded areas had more ammonium during the summer than uninvaded areas, while the opposite pattern occurred in the autumn.

These contrasting examples suggest that the dynamics of both the plant and microbial communities likely differ by site (Table 2). At the savanna site, it appears that any inorganic N produced is stored longer in the soil than at the other two sites. At the grassland site, net N mineralization rates are higher and inorganic N produced may be assimilated by the more densely populated *A. elatius* (e.g., during autumn tillering), leading to less inorganic N stored in the soil. In the case of both sites, stored inorganic N could be subject to other ecosystem fates with the addition of snowmelt or rainfall. Past research in California, U.S. grasslands highlighted the seasonal pattern of high nitrification rates with the transition from dry to wet seasons (see Parker and Schimel 2011; Xiang et al. 2008). Similarly, Yahdjian et al. (2010) reported pulses of higher net nitrification following significant rainfall events in Patagonian grasslands. Stored inorganic N may be subject to additional ecosystem fates that we did not measure, such as leaching or denitrification to the atmosphere. Of the three sites, it appears that the savanna has the greatest potential for significant episodic N transformations or stimulation of N loss with the addition of water.

An important question that follows from our observations at the grassland and shrubland sites is: Why is *A. elatius* associated with accelerated soil N cycling rates at some sites but not all? Although our study was not designed to test causality, we did measure several factors that could help to explain higher N cycling rates in invaded areas. We were interested in quantifying aboveground biomass production (stems and leaves), plus thatch, in invaded compared with uninvaded areas (e.g., Stanley et al. 2011). We were also interested in exploring whether *A. elatius* had higher aboveground biomass N content than that of grass tissues present in uninvaded areas, similar to invasive species in other grassland ecosystems (e.g., Rossiter-Rachor et al. 2009; James 2008). As demonstrated primarily through studies of N saturation in forested ecosystems (Aber et al. 1998), higher leaf and litter N content can result in accelerated N cycling processes. In addition, more thatch cover can alter soil moisture content by minimizing evaporative losses and holding more water locally (Liang et al. 2017), a particularly important control on tallgrass prairie species (Craine et al. 2010). Thatch cover can also limit rates of primary productivity in native tallgrass species by reducing photosynthetically active radiation to growing shoots and preventing cooling of emergent leaves (Knapp and Seastedt 1986).

Prior evidence that *A. elatius* produces more thatch than native prairie communities prompted an aboveground biomass harvest in September 2019. We measured approximately two-fold higher aboveground biomass at the grassland and shrubland sites in invaded compared with uninvaded areas. At the savanna, the difference between invaded and uninvaded areas was less dramatic, but the site exhibited the same pattern as the others, with invaded areas ~ 200 g m^−2^ greater in aboveground biomass than uninvaded areas (Fig. 2a). Stanley et al. (2011) also observed higher thatch production by *A. elatius* and other exotic species compared with native plant species in the Pacific Northwest (US). Interestingly, patterns in soil moisture during autumn did not differ substantially between invaded and uninvaded plots (Table 2, Fig. 1f). However, our snapshots of soil moisture during soil sampling are insufficient to determine its relationship with *A. elatius*; this question remains outstanding and requires further investigation.

We also considered the C:N ratios of aboveground biomass tissues (leaves, stems, and thatch), as an indication of whether *A. elatius* dominated vegetation is N-enriched relative to native prairie dominated vegetation. We measured a significant effect of site x invasion on aboveground biomass C:N (*p* = 0.015), however, only at the savanna did we find slightly lower C:N ratios in invaded compared with uninvaded areas. In all other cases, we observed the opposite pattern (Fig. 2b). Our results are challenging to interpret since our harvests in invaded areas were not 100% *A. elatius*; there were some non-native species present in uninvaded plots, and we sampled aboveground biomass after tissues had senesced. To better understand differences in aboveground biomass C:N ratios, it would be useful to compare tissue chemistry in live stems vs standing dead, and to analyze thatch separately.

In conclusion, we observed different relationships between *A. elatius* invasion and soil N cycling across sites in the Colorado foothills region. Similar complexity has been noted in studies evaluating the relationships between Cheatgrass (*Bromus tectorum*) invasion and nutrient cycling (Concilio et al. 2015). However, the differences observed in measured variables between invaded and uninvaded areas and among sites lead us to some important conclusions and logical next steps. First, the inconsistent directional difference between net N mineralization rates in invaded and uninvaded areas across sites suggests that while one might observe high-density *A. elatius* growth across the foothills of the Colorado Front Range, the relationship between soil N cycling and the invasive may be influenced by other factors that differ across locations. It is possible, for example, that rather than causing changes to soil N cycling, *A. elatius* exploits areas that have higher net N mineralization rates. A future study to evaluate causality of invasion and variation in soil N cycling rates would be useful. Second, understanding whether restoration objectives are feasible following widespread *A. elatius* invasion will be important. Many native grasses like Big Bluestem (*Andropogon gerardii*) are adapted to low-N soil environments (Averett et al. 2004). Thus, a logical next step is to determine whether native grass species of interest, such as *A. gerardii*, can persist in soils previously colonized by *A. elatius*. Such research will be important to inform how best to maintain or restore native plant community composition in tallgrass prairie ecosystems.

## Supplementary Information

Below is the link to the electronic supplementary material.

Supplementary file1 (DOCX 6452 KB)

## Data Availability

Data are provided as a supplement to this paper.

## Code availability

The code used in this study is available from the corresponding author on reasonable request.
