# Supplementary material for: Interactions between tall oatgrass invasion and soil nitrogen cycling
Source: Oecologia. 2022 Jun 7;199(2):419–26. doi: 10.1007/s00442-022-05192-x (PMC9226098; doi:10.1007/s00442-022-05192-x)
Supplement: Supplementary file 1 — Supplementary file1 (DOCX 6452 KB) [file 442_2022_5192_MOESM1_ESM.docx]

**Title:** Interactions between Tall Oatgrass Invasion and Soil Nitrogen Cycling

**Authors:** Eve-Lyn S. Hinckley^1,2*^, Hannah R. Miller^1,2^, Ann Lezberg^3^, and Brian Anacker^3^

**Affiliations:**

^1^Institute of Arctic and Alpine Research, Sustainability, Energy, and Environment Complex, 4001 Discovery Drive, Boulder, CO 80303

^2^Environmental Studies Program, University of Colorado, Sustainability, Energy, and Environment Complex, 4001 Discovery Drive, Boulder, CO 80303

^3^City of Boulder Open Space and Mountain Parks, 2520 55^th^ Street, Boulder, CO 80301

***Corresponding Author:** eve.hinckley@colorado.edu

### **Supplementary Material**

**Figure S1.** Site map with images from each sampling location. Inset shows the location of Colorado within the U.S.

*Structural Equation Model*

We used a structural equation model (SEM) fit with the cfa() function in the *R* package semTools to test two regressions: *A. elatius* as a predictor of vegetation, and, in turn, vegetation as a predictor of soil (Fig. S1). In Fig. S1, the relationships are depicted with black lines. Vegetation and soil (circles, Fig. S1) are unobserved latent variables. The latent variables are “indicated” by the measured variables (squares, Fig. S1) connected via gray paths. We weighted the lines by their coefficient size and the numbers next to the lines are standardized path coefficients (solid lines = positive coefficients and dashed lines = negative coefficients). Both summer and autumn seasons were included.

Our sample size was small (we fit 12 parameters with just 24 observations), and inclusion of the savanna site, which defied the patterns in the other two sites (see main text), would not allow our model to converge, so we eliminated it for this exercise. The model yielded *x^2^* = 4.9, *p* = 0.293, RMSEA = 0.999, and Comparative Fit Index (CFI) = 0.973. Ultimately, not all *r^2^* values could be estimated, so *r^2^* is not shown; this result is indicative of an overparameterized model. Thus, we include the SEM here to provide an alternative statistical analysis for our dataset but feel that the linear models reported in the main text are a better approach to use for interpreting our results.

###


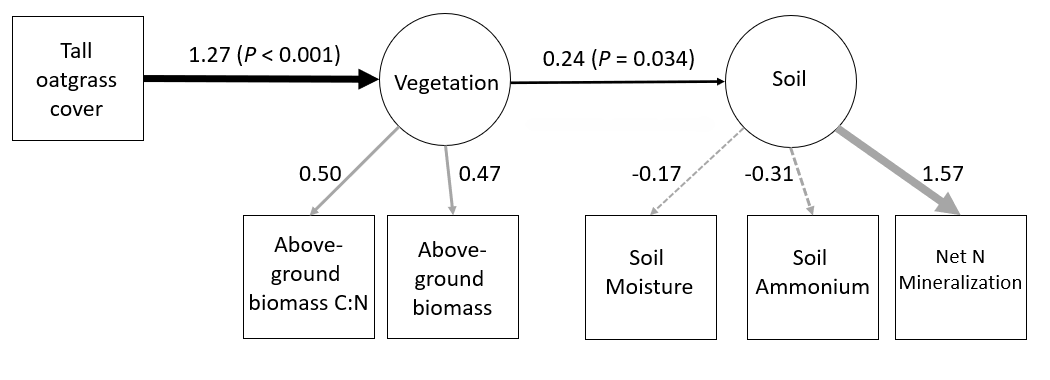


**Figure S2**. Structural Equation Model results. Units as written in the main text of the manuscript.

**Table S1**. Soil and plant data (units as described in the main text of the manuscript)

| **Season** | **Site** | **Plot** | **Invasion** | **TallOatgrassCover** | **Ammonium** | **Net_Min** | **SoilMoist** | **VegBiomass** | **VegBiomass_CN** |
| --- | --- | --- | --- | --- | --- | --- | --- | --- | --- |
| Summer | Shrubland | 1 | Uninvaded | 0 | 253.3573 | 0.078156 | 7.528314 | 454.476 | 45.5 |
| Summer | Shrubland | 2 | Uninvaded | 0 | 248.5546 | 7.095289 | 10.76775 | 361.4 | 48.66 |
| Summer | Shrubland | 3 | Uninvaded | 0 | 93.09494 | 16.93708 | 12.45855 | 911.36 | 41.28 |
| Summer | Shrubland | 4 | Invaded | 84 | 95.27604 | 51.74256 | 9.950915 | 1254.72 | 49.06 |
| Summer | Shrubland | 5 | Invaded | 40 | 42.26153 | 32.41965 | 18.26625 | 1711.68 | 48.56 |
| Summer | Shrubland | 6 | Invaded | 60 | 88.11712 | 114.3871 | 9.476955 | 997.6 | 43.55 |
| Summer | Grassland | 1 | Uninvaded | 0 | 162.7609 | 1.611257 | 20.04186 | 599.12 | 43.51 |
| Summer | Grassland | 2 | Uninvaded | 0 | 56.96379 | 21.74838 | 14.44261 | 373.84 | 32.32 |
| Summer | Grassland | 3 | Uninvaded | 0 | 70.68421 | 5.481291 | 15.98219 | 1300.8 | 35.35 |
| Summer | Grassland | 4 | Invaded | 82 | 130.7446 | 37.80379 | 20.04186 | 971.52 | 60.71 |
| Summer | Grassland | 5 | Invaded | 40 | 78.64441 | 22.84772 | 16.55209 | 696.48 | 46.8 |
| Summer | Grassland | 6 | Invaded | 75 | 135.811 | 47.44469 | 18.58691 | 1680.32 | 45.94 |
| Summer | Savanna | 1 | Uninvaded | 0 | 227.4379 | 41.67749 | 16.78467 | 607.68 | 56.28 |
| Summer | Savanna | 2 | Uninvaded | 0 | 28.55044 | 13.2192 | 18.62616 | 1844.88 | 68.73 |
| Summer | Savanna | 3 | Uninvaded | 0 | 48.32117 | 5.88521 | 20.73652 | 960.96 | 51.55 |
| Summer | Savanna | 4 | Invaded | 77 | 1469.553 | -27.5722 | 21.19937 | 1760.16 | 45.57 |
| Summer | Savanna | 5 | Invaded | 30 | 145.4996 | 10.33168 | 17.56075 | 1158.8 | 51.31 |
| Summer | Savanna | 6 | Invaded | 50 | 211.8994 | 5.404399 | 8.401977 | 1155.56 | 50.04 |
| Autumn | Shrubland | 1 | Uninvaded | 0 | 595.6919 | -16.1319 | 10.74751 | 454.476 | 45.5 |
| Autumn | Shrubland | 2 | Uninvaded | 0 | 179.853 | -0.54091 | 14.04847 | 361.4 | 48.66 |
| Autumn | Shrubland | 3 | Uninvaded | 0 | 193.7739 | 13.83525 | 15.5754 | 911.36 | 41.28 |
| Autumn | Shrubland | 4 | Invaded | 84 | 301.8045 | -4.04487 | 13.69261 | 1254.72 | 49.06 |
| Autumn | Shrubland | 5 | Invaded | 40 | 77.38641 | 5.8635 | 18.78896 | 1711.68 | 48.56 |
| Autumn | Shrubland | 6 | Invaded | 60 | 107.4846 | 13.70937 | 11.93451 | 997.6 | 43.55 |
| Autumn | Grassland | 1 | Uninvaded | 0 | 87.07405 | 3.8331 | 17.84703 | 599.12 | 43.51 |
| Autumn | Grassland | 2 | Uninvaded | 0 | 646.9713 | -18.1071 | 27.63571 | 373.84 | 32.32 |
| Autumn | Grassland | 3 | Uninvaded | 0 | 223.0055 | -13.4068 | 23.65226 | 1300.8 | 35.35 |
| Autumn | Grassland | 4 | Invaded | 82 | 154.4856 | 30.68522 | 27.25076 | 971.52 | 60.71 |
| Autumn | Grassland | 5 | Invaded | 40 | 120.2623 | 3.507472 | 25.78671 | 696.48 | 46.8 |
| Autumn | Grassland | 6 | Invaded | 75 | 136.5528 | 3.456324 | 18.49894 | 1680.32 | 45.94 |
| Autumn | Savanna | 1 | Uninvaded | 0 | 106.6145 | 4.892636 | 18.78914 | 607.68 | 56.28 |
| Autumn | Savanna | 2 | Uninvaded | 0 | 95.67889 | -1.64319 | 15.22556 | 1844.88 | 68.73 |
| Autumn | Savanna | 3 | Uninvaded | 0 | 51.96523 | 3.417246 | 21.6916 | 960.96 | 51.55 |
| Autumn | Savanna | 4 | Invaded | 77 | 346.5824 | -2.226 | 22.36136 | 1760.16 | 45.57 |
| Autumn | Savanna | 5 | Invaded | 30 | 187.5487 | 9.184929 | 15.22298 | 1158.8 | 51.31 |
| Autumn | Savanna | 6 | Invaded | 50 | 715.6538 | -3.31132 | 14.17625 | 1155.56 | 50.04 |
